# A prospective analysis of physical activity and mental health in children: the GECKO Drenthe cohort

**DOI:** 10.1186/s12966-023-01506-1

**Published:** 2023-09-25

**Authors:** Lu Yang, Eva Corpeleijn, Esther Hartman

**Affiliations:** 1 Department of Human Movement Sciences, University Medical Center Groningen, University of Groningen, Groningen, The Netherlands; 2 Department of Epidemiology, University Medical Center Groningen, University of Groningen, Groningen, The Netherlands

**Keywords:** Physical activity, Sedentary behaviors, Mental health, Children, Cohort study

## Abstract

**Background:**

Mental health problems in young people have become a global health burden. The positive effects of physical activity on mental health in adults are well known but still not clear in children. The aim of this study was to investigate to what extent physical activity in early childhood would affect mental health in middle childhood.

**Methods:**

From the Dutch GECKO Drenthe birth cohort, 850 children (51.5% boys) were enrolled in this analysis. Physical activity and sedentary time were measured at age 5–6 using ActiGraph GT3X. Mental health was assessed using the Strengths and Difficulties Questionnaire (SDQ) at age 5–6 and age 10–11. Multiple linear regression models were used to estimate the associations between physical activity, sedentary time and SDQ subscales, stratified by gender, adjusting for age, BMI, maternal education level, family size, accelerometer wear time and season, and additionally adjusting for SDQ scores at age 5–6 to take tracking of mental health over time into account.

**Results:**

Greater physical activity volume at age 5–6 was associated with lower peer problems scores at age 10–11 in boys and girls. An increase in MVPA was associated with lower peer problems scores in boys (b = -0.445, -0.713 to -0.176) and girls (b = -0.354, -0.601 to -0.107), however, increased sedentary time was linked to higher peer problems scores in boys (b = 1.18, 0.455 to 1.906) and girls (b = 0.870, 0.191 to 1.550). For hyperactivity, higher levels of physical activity volume and MVPA were associated with higher hyperactivity scores in boys. Increased sedentary time was related to lower hyperactivity scores in boys. Further adjustment for SDQ scores at age 5–6 attenuated associations between physical activity and hyperactivity in boys but hardly changed the relationships with peer problems. No significant associations between physical activity and other SDQ subscales or total difficulties scores were observed, neither in boys nor in girls.

**Conclusions:**

Children who are more physically active at age 5–6 have fewer peer problems at age 10–11, and for boys, greater activity levels at age 5–6 could be an indicator of hyperactivity at age 10–11.

**Supplementary Information:**

The online version contains supplementary material available at 10.1186/s12966-023-01506-1.

## Introduction

Mental health problems affect 10–20% of children and adolescents worldwide, and these problems can continue beyond childhood and adolescence [1]. The concept of mental health can be defined as a multidimensional state of well-being, with both negative indicators such as symptoms of depression, anxiety or behavioral problems, and positive indicators such as self-concept [2]. Almost half of the mental disorders in adults were reported to emerge before 14 years of age [3], which emphasizes the need to prevent and manage mental health problems in childhood.

A growing body of evidence shows that physical activity may be associated with mental health outcomes [4, 5]. Three mechanisms have been most reported to explain the relationships between physical activity and mental health. First, at the neurobiological level, participation in physical activity is believed to enhance cognition and mental health by changing the structural and functional composition of the brain [6, 7]. Furthermore, physical activity promotes the release of endorphins in the brain, which can ease pain and produce feelings of euphoria, and hence reduces depression and increases well-being [8]. Second, another hypothesis proposes that physical activity mediates mental health outcomes by changing behaviors. For example, physical activity could improve sleep volume and pattern in children by increasing energy expenditure, and then reduce cognitive deficits caused by insufficient sleep [9]. Third, physical activity has the potential to improve well-being by satisfying basic psychological needs for social connectedness, self-acceptance and life purposes [9].

As physical activity is a modifiable factor, it may play a role in the prevention and management of mental problems. Systematic review studies have found positive effects of physical activity interventions on mental health in different age groups. Peng et al. reported that the administration of a physical activity intervention may lead to moderate improvements in symptoms of depression in adolescents aged 12–18 years old [10]. In addition, Hale et al. summarized studies involving physical activity interventions for children aged 6 to 11 years old and concluded that these interventions had positive effects on mental health in school and neighborhood settings, but evidence on reducing ill-being in children remained unclear [11].

Although various studies have suggested that physical activity may promote various mental health outcomes in children, findings about the impacts of physical activity on young children were not consistent. A systematic review of observational studies in children aged 0–5 years found that clear conclusions about the associations between physical activity and mental health cannot be drawn because too few studies existed [12]. Researchers also pointed out that a critical limitation of those included studies was the lack of device-based physical activity [12], which may have resulted in the inconsistent results in young children. The researchers warranted more studies using device-based physical activity to improve research quality. More recently, a review study aiming to determine the effects of physical activity on mental health outcomes in preschoolers, children and adolescents found significant effects of physical activity interventions on mental health in adolescents aged 12–18 but not in children aged 6–11. This reivew also reported that evidence for physical activity effects on mental health of preschoolers was nearly non-existent [13]. Most of involved studies in this review were about adolescents, which to extent explains the inconsistent results in children and limited evidence in preschoolers. Therefore, there is a need for studies on young children to determine the associations between physical activity and mental health during this period.

Given that a variety of developmental changes are happening in the brain during the early stages of human life [14], identifying the effects of physical activity in the early stage on the later psychological outcomes may further clarify the role of physical activity in the development of psychological health. Therefore, the purpose of this study is to explore whether device-based physical activity in early childhood is associated with mental health in later childhood.

## Methods

### Study design

The GECKO Drenthe study is a population-based birth cohort focusing on early risk factors for children’s physical and mental health. In 2006, almost 3000 pregnant women were recruited, and their children were monitored from the last trimester until now. More details of the GECKO cohort study have been described elsewhere [15]. For the current study, of 1389 children with accelerometer-derived physical activity data measured at age 5–6, 1070 children with valid physical activity data were enrolled. Among these, children who had valid data on mental health at age 10–11 were included in the main analysis (n = 850). This study was approved by Medical Ethics Committee of the University Medical Center Groningen and performed in accordance with the Declaration of Helsinki. Informed consent of participation was given by parents or guardians. The cohort is registered on www.birthcohorts.net (id 138).

### Physical activity

The ActiGraph GT3X accelerometer (ActiGraph, Pensacola, Florida, USA) was used to measure sedentary time and physical activity. The accelerometer was placed on the right hip and worn during waking hours for four days including at least one weekend day, except for water-based activities such as swimming and bathing. Data were collected using a frequency of 30 Hz and analyzed with a 15-s epoch. Non-wear time of the ActiGraph was classified as a minimum length of 90 min, with allowance of 2 min intervals of spike tolerance with the up/downstream 30-min consecutive zero counts window [16]. Physical activity volume was computed as total accelerometer counts (counts per minute (cpm)) on average per day. Physical activity intensity was computed using cut-off points recommended by Butte et al*.*, i.e. sedentary time (≤ 819 cpm), light physical activity (LPA, 820–3907 cpm), moderate physical activity (MPA, 3908–6111 cpm), vigorous physical activity (VPA, ≥ 6112 cpm), and moderate-to-vigorous physical activity (MVPA, ≥ 3908 cpm) [17]. Time spent in different physical activity intensity was used in analyses. A valid wearing period was selected as being from 6:00 until midnight. A valid measurement was defined as a wear time of at least 600 min/day for at least three days regardless weekday or weekend. Season was also obtained from the ActiGraph output. Winter was defined as December – February, spring as March – May, summer as June – August and autumn as September – November.

### Mental health

The Dutch version of the Strengths and Difficulties Questionnaire (SDQ) was used to measure mental health of children at age 5–6 and age 10–11 (Goodman, 1997). SDQ is a screening tool for identifying mental problems that was filled in by parents in the present study. It has shown adequate reliability and validity from preschoolers to adolescents [18, 19]. The SDQ is a short questionnaire that comprises 5 subscales: (1) hyperactivity/inattention, (2) behavioral problems, (3) peer problems, (4) emotional problems, and (5) prosocial behaviors. Each subscale consists of 5 items on a 3-point scale, namely 0 (not true), 1 (somewhat true), and 2 (certainly true). The scores for each subscale can range from 0 to 10 if all items are completed. The subscale scores can only be scaled up pro-rata if at least 3 items are completed. Otherwise, the subscale scores are set to missing [3]. In the present study, a valid measurement of mental health was defined as no missing scores for hyperactivity, behavior problems, peer problems and emotional problems at age 10–11. Total difficulties scores are calculated by summing scores from all subscales except prosocial behaviors subscale. Externalizing problems scores are calculated as the sum of hyperactivity and behavior problems subscales, and internalizing problems scores are the sum of emotional and peer problems subscales.

### Other factors

Exact ages of children at physical activity and SDQ measurement were collected by questionnaires filled in by parents. Height, weight at the age of 5–6 and 10–11 years were measured by trained nurses according to standardized protocols, as described previously [20]. Body mass index (BMI) was calculated accordingly. With regard to socio-economic status, maternal education level was obtained from questionnaires filled in by the parents during pregnancy and classified into the following groups: (1) no education or lower general secondary education, (2) senior secondary vocational education or higher general secondary education/pre-university education, and (3) higher vocational education or university level. Family size, including number of adults and number of children in a household, was collected using questionnaire filled in by parents when the child was born.

### Statistical analysis

As sex differences in physical activity and SDQ scores were observed in children at age 5–6 in our previous study, the current analysis were separately performed for boys and girls [21]. Sample descriptive characteristics (sex, age, BMI, family size, maternal education) were expressed as frequency, percentage of total number for categorical variables, mean and SD for normally distributed variables, and median with interquartile range (25^th^-75^th^ percentile) for non-normally variables. The differences between boys and girls on physical activity and SDQ were compared using independent Student’s t-tests and non-parametric Mann–Whitney U tests.

Spearman’s rank correlations were calculated to evaluate the association between physical activity at age 5–6 and SDQ scores at age 10–11. Next, multiple linear regression models were fitted for SDQ subscales showing significant correlations (*p* < 0.05). The unit of physical activity was 100 cpm for physical activity volume, 30 min for sedentary time and light physical activity, and 10 min for MVPA. As determinants, physical activity volume, sedentary time and MVPA needed log-transformation with base 2 to obtain normal distribution of residuals, before regression models were constructed. Missing values in BMI and family size were replaced with the variable mean. In the regression analysis, model 1 was defined as the crude model. Model 2 was adjusted for potential confounders including exact age at SDQ measurement, BMI at age 10–11, family size, maternal education, accelerometer wear time and season. Then, to investigate to what extent tracking of mental health over time was relevant for the findings, model 2 + was adjusted for SDQ scores at age 5–6 in addition to model 2. Outcomes were presented as b coefficients, standardized b coefficients and 95% CI. To enhance the robustness of our findings, we performed a sensitivity analysis by repeating the linear regression analysis on a restricted sample of children who provided valid accelerometry data during both weekdays and at least one weekend.

All statistical analyses were performed using IBM SPSS Statistics Version 28.0 (SPSS Inc., Chicago, IL). A 2-sided statistical significance was set at *P* < 0.05 for all analyses.

## Results

### Sample characteristics

**Fig. 1:**
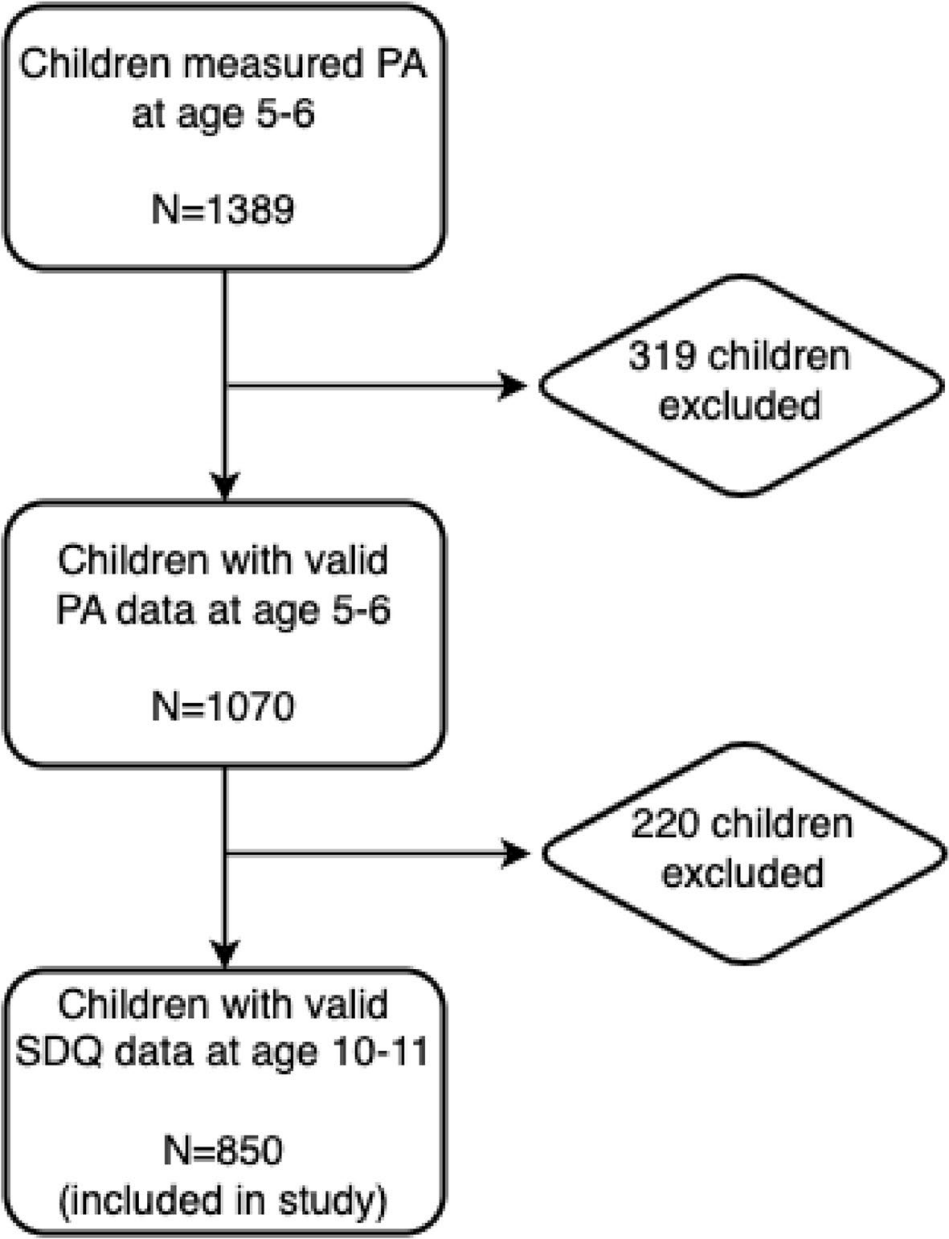
Flowchart of subject enrollment

Of 1070 children with valid physical activity data, 850 provided a valid SDQ questionnaire at age 10–11, including 438 boys and 412 girls (Fig. 1). Table 1 shows the differences between included and excluded participants. The descriptive characteristics and physical activity behaviors for involved boys and girls are displayed in Table 2. The mean age at which physical activity was assessed was 5.11 ± 0.89 years, whereas the mean ages for the initial and subsequent measurements of SDQ were 5.88 ± 0.39 years and 10.57 ± 0.55 years, respectively. Physical activity data indicated that total wear time in boys and girls was comparable, but boys were more active than girls at the age of 5–6 years. Boys spent less time in sedentary behaviors (*P* = 0.004), but more time in MPA (*P* < 0.001) and VPA (*P* < 0.001) than girls.

**Table 1:** Characteristics of excluded and included subjects

	**Excluded (*n* = 220)**	**Included (*n* = 850)**	***P* value**
**Sex **^**a**^			
Boys	114	438	0.94
Girls	106	412	
Age at physical activity measurement ^b^	5.11 (0.90)	5.10 (0.90)	0.14
Age at SDQ measurement ^b,d^	10.92 (0.53)	10.57(0.55)	< 0.001
**physical activity variables**			
physical activity volume (cpm) ^c^	1319.50 [1126.68, 1521.21]	1319.67 [1142.81,1517.29]	0.99
sedentary time (min) ^c^	369.32 [333.15, 407.70]	372.42 [334.80, 408.27]	0.82
LPA (min) ^b^	265.44 (36.63)	264.71 (38.20)	0.47
MPA (min) ^c^	43.81 [33.77, 54.84]	43.75 [34.81, 54.80]	0.80
VPA (min) ^c^	17.40 [11.22, 24.91]	16.56 [11.23, 24.19]	0.88
MVPA (min) ^c^	62.81 [45.95, 78.61]	61.25 [47.90, 80.08]	0.91

*SDQ* the Strengths and Difficulties Questionnaire, *LPA* Light Physical Activity, *MPA* Moderate Physical Activity, *VPA* Vigorous Physical Activity, *MVPA* Moderate to Vigorous Physical Activity

^a^ Chi-squared test

^b^ Independent samples t-test. Data was shown as mean (SD)

^c^ Non-parametric Mann–Whitney U test. Data was shown as median [P25, P75]

^d^ Age at 2^nd^ SDQ in excluded subjects *n* = 116 due to missing data

**Table 2:** Descriptive characteristics of the study population and physical activity variables

	**Boys**		**Girls**		***P* value boys *vs* girls**
	**N (%)**	**Mean (± SD)**	**N (%)**	**Mean (± SD)**	
Sex	438 (51.5%)		412(48.5%)		
Age at 1^st^ SDQ^a^		5.89 (± 0.39)		5.87 (± 0.38)	0.672
Age at 2^nd^ SDQ^b^		10.63 (± 0.55)		10.50 (± 0.54)	0.402
Age at physical activity measurement		5.11 (± 0.89)		5.09 (± 0.92)	0.059
BMI at age 10–11		17.41 (± 2.41)		17.65 (± 2.48)	0.107
Number of adults in the household					
1	6 (0.7%)		8 (0.9%)		
2	421 (49.8%)		389 (46.0%)		
3	0		2 (0.2%)		
4	2 (0.2%)		0		
Number of children in the household					
1	156 (18.4%)		156 (18.4%)		
2	195 (23.0%)		167 (19.7%)		
3	70 (8.3%)		63 (7.4%)		
4	11 (1.3%)		16 (1.9%)		
5	4 (0.5%)		4 (0.5%)		
6	0		1 (0.1%)		
Maternal education^c^					
1	195 (23.6%)		172 (20.8%)		
2	136 (16.4%)		129 (15.6%)		
3	97 (11.7%)		98 (11.9%)		
**physical activity variables**		**Median [P25, P75]/Mean (± SD)**		**Median [P25, P75]/Mean (± SD)**	
physical activity volume (cpm)		1362.40 [1209.10, 1575.16]		1249.40 [1079.65, 1457.77]	< 0.001
Total wear time (min)		701.31 [680.55, 724.00]		699.56 [673.59, 725.64]	0.060
Sedentary time (min)		368.00 [329.51, 402.47]		377.63 [340.46, 413.00]	0.004
LPA (min)		264.00 (± 36.39)		265.81 (± 40.00)	0.050
MPA (min)		47.17 [39.53, 60.07]		40.08 [31.39, 49.32]	< 0.001
VPA (min)		18.50 [12.46, 26.44]		14.50 [10.50, 21.92]	< 0.001
MVPA (min)		68.13 [53.03, 85.64]		54.44 [42.13, 71.82]	< 0.001

*SDQ* the Strengths and Difficulties Questionnaire, *LPA* Light Physical Activity, *MPA* Moderate Physical Activity, *VPA* Vigorous Physical Activity, *MVPA* Moderate to Vigorous Physical Activity

^a^ 1st SDQ: SDQ measured at age 5–6

^b^ 2^nd^ SDQ: SDQ measured at age 10–11

^c^ Maternal education: 1: no education or lower general secondary education; 2: senior secondary vocational education or higher general secondary education /pre-university education, and 3: higher vocational education or university level

An overview of SDQ scores for children aged 5–6 and age 10–11 is shown in Table 3. For children at age 10–11, boys scored higher than girls on total difficulties and externalizing problems. No significant differences in internalizing problems were observed. For SDQ subscales measured at age 10–11, scores on hyperactivity, behavioral problems and peer problems were higher for boys than girls (*p* < 0.01). Inversely, girls had higher SDQ scores on prosocial behaviors (*p* < 0.001). No significant difference in emotional problems between boys and girls was observed.

**Table 3:** Mental health: Overview of SDQ scores at age 5–6 and age 10–11

	**Boys**	**Girls**	***P* value**
	**Median [P25, P75]**	**Median [P25, P75]**	**boys *vs* girls**
**SDQ scores at age 10–11**			
Total difficulties	6 [3, 10]	4 [2, 7]	< 0.001
Externalizing	4 [2, 7]	2 [0, 4]	< 0.001
Internalizing	2 [0, 4]	2 [0, 4]	0.32
Hyperactivity	3 [1, 5]	1 [0, 3]	< 0.001
Behavior problems	0 [0, 1]	0 [0, 1]	< 0.001
Peer problems	0 [0, 2]	0 [0, 1]	0.013
Emotional problems	1 [0, 2]	1 [0, 3]	0.932
Prosocial behaviors	9 [8, 10]	10 [8, 10]	< 0.001
**SDQ scores at age 5–6**			
Total difficulties	5 [3, 8]	4 [2, 6]	< 0.001
Externalizing	3 [2, 6]	2 [1, 4]	< 0.001
Internalizing	1 [0, 3]	1 [0, 3]	0.510
Hyperactivity	3 [1, 4.75]	1 [0, 3]	< 0.001
Behavior problems	1 [0, 2]	0 [0, 1]	< 0.001
Peer problems	0 [0, 1]	0 [0, 1]	0.477
Emotional problems	1 [0, 2]	1 [0, 2]	0.229
Prosocial behaviors	9 [8, 10]	9 [8, 10]	< 0.001

*SDQ* the Strengths and Difficulties Questionnaire

### Associations between physical activity and SDQ

The correlations between physical activity at age 5–6 and SDQ at age 10–11 are presented in Table 4. No significant association between physical activity and total difficulties scores was observed, neither in boys nor in girls. For boys, higher levels of physical activity volume and intensity, including MPA, VPA and MVPA were significantly associated with lower internalizing scores and with higher externalizing scores. LPA was only negatively associated with internalizing scores. In contrast, increased sedentary time was associated with higher internalizing scores and lower externalizing scores. Regarding SDQ subscales, more physical activity volume and physical activity intensity (MPA, VPA, MVPA) were associated with higher hyperactivity scores and lower peer problems scores, while increased sedentary time was linked to lower hyperactivity scores and higher peer problems scores. The trends of correlations between physical activity, sedentary time and SDQ subscales in girls were similar to those observed in boys, but the correlations in girls were weaker and less often significant. In both boys and girls, no significant relationships of physical activity with behavioral problems, emotional problems and prosocial behaviors were observed. Results of Table 4 suggested the need for further analysis on subscale scores instead of total difficulties scores, externalizing or internalizing scores.

**Table 4:** Correlations between physical activity at age 5–6 and SDQ scores at age 10–11

	**Total Difficulties**	**Internalizing**	**Externalizing**	**Hyperactivity**	**Behavioral problems**	**Peer problems**	**Emotional problems**	**Prosocial behaviors**
***Boys***								
Physical activity volume (cpm)	0.038	-0.098^*^	0.131^**^	0.141^**^	0.062	-0.163^**^	-0.023	0.010
Sedentary time (min)	-0.024	0.104^*^	-0.121^*^	-0.135^**^	-0.031	0.159^**^	0.035	-0.024
LPA (min)	-0.036	-0.111^*^	0.033	0.052	-0.018	-0.119^*^	-0.077	0.032
MPA (min)	0.059	-0.095^*^	0.161^**^	0.170^**^	0.084	-0.138^**^	-0.029	-0.045
VPA (min)	0.036	-0.094^*^	0.120^*^	0.127^**^	0.060	-0.156^**^	-0.018	0.018
MVPA (min)	0.054	-0.097^*^	0.153^**^	0.160^**^	0.082	-0.155^**^	-0.020	-0.013
***Girls***								
Physical activity volume (cpm)	0.024	-0.078	0.087	0.096	0.031	-0.112^*^	-0.020	-0.046
Sedentary time (min)	-0.053	0.036	-0.099^*^	-0.091	-0.084	0.061	-0.008	0.059
LPA (min)	0.035	-0.022	0.076	0.070	0.045	-0.048	0.016	0.029
MPA (min)	0.039	-0.077	0.114^*^	0.128^**^	0.030	-0.079	-0.029	-0.030
VPA (min)	-0.011	-0.106^*^	0.049	0.072	-0.022	-0.141^**^	-0.054	-0.015
MVPA (min)	0.013	-0.104^*^	0.092	0.110^*^	0.009	-0.129^**^	-0.045	-0.030

^**^P < 0.01, ^*^P < 0.05; *SDQ* the Strengths and Difficulties Questionnaire, *LPA* Light Physical Activity, *MPA* Moderate Physical Activity, *VPA* Vigorous Physical Activity, *MVPA* Moderate to Vigorous Physical Activity

Table 5 presents the adjusted associations between physical activity and peer problem scores in boys and girls. It shows that overall, higher levels of physical activity at age 5–6 were related to fewer peer problems score at age 10–11 in boys and girls. In more detail, model 2 demonstrated that more physical activity volume was associated with lower peer problems scores for boys (b = -0.232, -0.371 to -0.093) and girls (b = -0.189, -0.314 to -0.063). Higher levels of MVPA were also associated with lower peer problems scores for boys (b = -0.445, -0.713 to -0.176) and girls (b = -0.354, -0.601 to -0.107). The relationships between MPA, VPA and peer problems paralleled the associations observed between MVPA and peer problems (See Supplementary Table S1, Additional File 1). In contrast, an increase in sedentary time was associated with higher peer problems scores in boys (b = 1.18, 0.455 to 1.906) and girls (b = 0.870, 0.191 to 1.550). Comparison of standardized b indicates that these associations are stronger in boys than in girls. Model 2 + in Table 5 indicates that the additional adjustment for peer problems scores measured at age 5–6 hardly changed the associations between physical activity and peer problems scores at age 10–11.

**Table 5:** Multiple linear regression analysis for physical activity at age 5–6 and SDQ subscale scores at age 10–11

	**Boys**			**Girls**		
	**B**	**Std. B**	**95% CI of B**	**B**	**Std. B**	**95% CI of B**
***Peer problems***						
**Physical activity volume (100 cpm)**						
Model 1	-0.199**	-0.142	(-0.331 to -0.067)	-0.174**	-0.139	(-0.295 to -0.052)
Model 2	-0.232**	-0.166	(-0.371 to -0.093)	-0.189**	-0.152	(-0.314 to -0.063)
Model 2 + peer problems at age 5	-0.229**	-0.164	(-0.371 to -0.087)	-0.163*	-0.134	(-0.291 to -0.035)
**MVPA (per 10 min)**						
Model 1	-0.408**	-0.149	(-0.667 to -0.150)	-0.359**	-0.146	(-0.599 to -0.119)
Model 2	-0.445**	-0.162	(-0.713 to -0.176)	-0.354**	-0.144	(-0.601 to -0.107)
Model 2 + peer problems at age 5	-0.493**	-0.179	(-0.767 to -0.219)	-0.315*	-0.128	(-0.570 to -0.059)
**LPA (per 30 min)**						
Model 1	-0.074	-0.062	(-0.187 to 0.039)	-0.055	-0.054	(-0.153 to 0.044)
Model 2	-0.097	-0.082	(-0.213 to 0.019)	-0.083	-0.083	(-0.189 to 0.022)
Model 2 + peer problems at age 5	-0.065	-0.054	(-0.184 to 0.055)	-0.059	-0.058	(-0.167 to 0.049)
**Sedentary time (per 30 min)**						
Model 1	0.807*	0.122	(0.181 to 1.434)	0.596	0.095	(-0.020 to 1.212)
Model 2	1.180**	0.178	(0.455 to 1.906)	0.870*	0.138	(0.191 to 1.550)
Model 2 + peer problems at age 5	1.053**	0.159	(0.308 to 1.799)	0.733*	0.119	(0.037 to 1.429)
***Hyperactivity***						
**Physical activity volume (per 100 cpm)**						
Model 1	0.290*	0.108	(0.036 to 0.544)	0.152	0.072	(-0.057 to 0.361)
Model 2	0.350**	0.130	(0.088 to 0.612)	0.127	0.060	(-0.091 to 0.346)
Model 2 + hyperactivity at age 5	0.069	0.027	(-0.160 to 0.298)	-0.115	-0.055	(-0.297 to 0.067)
**MVPA (per 10 min)**						
Model 1	0.764**	0.145	(0.269 to 1.259)	0.303	0.072	(-0.111 to 0.718)
Model 2	0.893**	0.170	(0.390 to 1.396)	0.324	0.077	(-0.105 to 0.753)
Model 2 + hyperactivity at age 5	0.347	0.068	(-0.096 to 0.790)	-0.187	-0.045	(-0.549 to 0.175)
**LPA (per 30 min)**						
Model 1	0.111	0.049	(-0.106 to 0.327)	0.120	0.070	(-0.049 to 0.288)
Model 2	0.147	0.065	(-0.070 to 0.365)	0.069	0.040	(-0.114 to 0.252)
Model 2 + hyperactivity at age 5	0.031	0.014	(-0.158 to 0.219)	0.037	0.022	(-0.116 to 0.190)
**Sedentary time (per 30 min)**						
Model 1	-1.553*	-0.122	(-2.755 to -0.352)	-0.950	-0.088	(-2.006 to 0.107)
Model 2	-1.884**	-0.148	(-3.251 to -0.517)	-0.590	-0.055	(-1.770 to 0.590)
Model 2 + hyperactivity at age 5	-0.746	-0.061	(-1.947 to 0.455)	0.154	0.015	(-0.829 to 1.138)

^**^P < 0.01, *P < 0.05. Model 1: crude model; Model 2: the primary adjusted model, adjusting for age, BMI, family size, maternal education, wear time and season; Model 2 + peer problems/hyperactivity at age 5: further adjusting for SDQ scores at age 5 in addition to model 2. *SDQ* the Strengths and Difficulties Questionnaire, *LPA* Light Physical Activity, *MVPA* Moderate to Vigorous Physical Activity

Table 5 also shows that higher levels of physical activity at age 5–6 were related to higher hyperactivity scores at age 10–11 in boys, but no such relationship was observed in girls. For boys, model 2 demonstrated that increased physical activity volume and MVPA were associated with higher hyperactivity scores. Likewise, increased MPA and VPA were also related to higher hyperactivity scores (See Supplementary Table S1, Additional File 1). Inversely, a rise of sedentary time was associated with lower hyperactivity scores (b = -1.884, -3.251 to -0.517). The additional adjustment for hyperactivity scores at age 5–6 attenuated all associations between physical activity and hyperactivity scores (Table 5). For girls, no significant associations between physical activity and hyperactivity were observed. No significant associations between physical activity and other SDQ subscales were observed, neither in boys nor girls (See Supplementary Table S2, Additional File 1).

In the sensitivity analyses, the main associations between physical activity volume, MVPA and peer problems scores remained consistent across genders. However, the significant relationship with sedentary time was only observed in boys. Additionally, the associations between physical activity volume, MVPA, sedentary time and hyperactivity scores in boys also remained consistent in the sensitivity analyses (See Supplementary Table S3, Additional File 1).

## Discussion

The aim of the present study was to investigate whether device-based physical activity in early childhood is associated with mental health in middle childhood. The findings showed that higher levels of physical activity at age 5–6 were related to lower peer problems scores at age 10–11 years in both boys and girls. In contrast, increased sedentary time at age 5–6 was associated with higher peer problems scores at age 10–11. Further adjustment for mental health scores at age 5–6 hardly change the associations between MVPA and peer problems. For hyperactivity, increased MVPA and decreased sedentary time were associated with higher hyperactivity scores in boys, but not in girls. These associations were attenuated after adjustment for hyperactivity scores at age 5–6.

Our findings regarding the association between MVPA and peer problems in boys was strongly supported by a UK longitudinal study, which found that higher levels of device-based MVPA in boys at age 7 were associated with less peer problems at age 11 [22]. The present study utilized device-based measurements of physical activity at 5–6 years old, thereby extending the existing evidence that physical activity at a younger age serves as a predictor of peer problems at ages 10–11. The association between MVPA and peer problems in boys might be explained by psychosocial factors. Participation in sports could offer opportunities for developing social skills and improving social competence, which would in turn help to reduce the likelihood of the development of peer problems [23]. Moreover, it is commonly observed that children tend to gravitate towards peers who exhibit similar behaviors and distance from those who behave differently [24]. A previous study has shown that it was possible that some boys who did not participate in sports like their peers, may experience isolation from them, which could be perceived as having social difficulties with peers [25]. This, therefore, probably explains why engaging in MVPA could have a positive impact on boys’ peer relationships in our study.

An intriguing finding in our study is the correlation between MVPA and peer issues in girls. To the best of our knowledge, this is the first study that shows the positive impact of MVPA at age 5–6 on girls' peer relationships at age 10–11. Ahn et al. have previously reported that light physical activity in girls aged 7 was associated with decreased peer problems at age 11 [22]. The discrepancy in results could be attributed to different cut-off points used to define sedentary time, light physical activity and MVPA, as well as the different ages of the participants. Our finding indicates that MVPA at an early age could have positive impact on girls’ peer relationships in middle childhood, offering evidence for supporting young girls to engage in sports and physical activity from early childhood onwards. A previous study has shown that some girls may feel less support from parents or teachers in physical activity pursuits than boys [26]. This lack of support could stem from the bias that girls are not as capable in sports as boys [26]. When girls do not have enough support, they may be less likely to participate in physical activity and hence miss out on the benefits of physical activity. By providing evidence that girls could benefit from MVPA in similar ways to boys, our study might help to dispel sex-based stereotype about physical activity and promote equal opportunities for girls to enjoy MVPA from an early age.

An interesting observation in this study is that the associations between physical activity, sedentary time and peer problems were more consistent in boys than in girls. This difference may be driven by different expectations for physical activity participation among boys and girls, which are shaped by societal expectations and gender norms. In many cases, athletic ability and sport participation are seen as important criteria for popularity and social recognition among boys, while these factors are less emphasized for girls [26]. As a result, boys may place a higher value on physical activity and be more motivated to participate in high levels of physical activity to shape their peer relationships, whereas girls may not feel the same pressure to do so. Furthermore, the stronger correlation between sedentary time and peer problems in boys compared to girls may be influenced by varying sedentary pursuits. Productive sedentary time (e.g., reading, doing homework) and non-productive sedentary time (e.g., watching TV) have often been grouped together, despite potentially having different impacts on physical and mental health [27, 28]. A prior study found that while boys were less sedentary than girls, they had more screen time [29], which could also have been the case in the current study. The previous study indicated a higher possibility of peer problems in children with more screen time [30], but there is a lack of evidence for the effects of other sedentary behaviors on peer problems. Hence, future research on sedentary pursuits is therefore warranted to better understand how different sedentary behaviors can impact mental health.

With respect to hyperactivity, our results were in accordance with a UK longitudinal study using device-based physical activity [22], but also opposed to several studies indicating that self- or parent-reported physical activity could be a protective factor against hyperactive symptoms [31, 32]. These previous studies relied on questionnaires, instead of accelerometers, to evaluate participation of physical activity, which to an extent explains the contradiction with our findings. Self- and parent-reported questionnaires could mainly capture organized and structured physical activity, such as participation in organized sport. In contrast, accelerometers can also detect spontaneous movements, which are often associated with neurodevelopmental disorder such as attention deficit/hyperactivity disorder (ADHD) [33]. According to a meta-analysis, children with ADHD exhibited higher activity levels compared to typically developing children, underscoring the value of actigraph as a tool for monitoring ambulatory activity in ADHD cases [34]. This higher activity level in children could result in an overestimation of physical activity when assessed using accelerometers. Consequently, the device-based physical activity would not serve as a protective factor against hyperactive symptoms in this study. Moreover, in studies where physical activity volume and intensity were reported via questionnaires, it is important to consider that individuals might encounter challenges in accurately recalling or assessing physical activity levels and intensity. This potential discrepancy could lead to measurement bias and yield dissimilar outcomes compared to device-based assessments.

We have previously shown in a cross-sectional analysis that more MVPA and less sedentary time were associated with higher hyperactivity scores in children aged 5–6 [21]. To examine how the tracking of SDQ over time relates to the current findings, model 2 was additionally adjusted for SDQ subscale scores measured at age 5–6. As a result, the associations with hyperactivity were attenuated. The most likely interpretation for this phenomenon is that the association between physical activity and hyperactivity was already present at 5–6 years of age, and that this association tracks with age. The estimates for sedentary time and MVPA are halved but remain significant. This indicates that the association has become even stronger than at 5–6 years of age. It also indicates that hyperactivity may be an individual trait that can persist from preschool years into middle childhood and can be measured using accelerometry [34]. In sensitivity analyses, the associations with hyperactivity at age 10–11 remained unchanged after the additional adjustment. This reinforces the associations in children who consistently maintain higher physical activity level throughout the week, supporting the idea of physical activity as an indicator of hyperactivity in later childhood. Using the same approach, however, the associations between physical activity at age 5–6 and peer problems at age 10–11 remained nearly unchanged. For peer problems, it is likely that low physical activity at 5–6 years of age indeed increases the risk of developing peer problems later in life, whereas the reverse scenario, that physical activity is affected by peer problems at age 5–6 years of age, is highly unlikely. This may indicate a potential causal, albeit weak relationship between being physically active in early life and developing healthy peer relationships in middle childhood.

Some previous studies have reported significant associations between physical activity, sedentary time and SDQ total difficulties scores, however, no such association was observed in the current study [3, 22]. Total difficulties scores are the sum of internalizing and externalizing subscales. Our data showed that the relationships with the internalizing and externalizing subscales were in opposite directions, which may result in the neutralization of the association between physical activity and total difficulties scores. Therefore, we recommend for future studies to conduct separate analyses of data related to internalizing and externalizing subscales instead of solely focusing on total difficulties scores.

The key strengths of this study include the device-based physical activity in very young children and the longitudinal study design. The use of device-based measurement for physical activity is relatively rare in preschoolers, with most studies relying on questionnaires to assess physical activity in this age group [13]. The current study extended the accelerometer usage into the 5–6-year-olds. Furthermore, by using physical activity at age 5–6 and SDQ at age 10–11 and controlling for previous SDQ at age 5–6, this longitudinal study provides additional insight into the effects of physical activity on children’s mental health over time. However, one limitation of this study is that physical activity data was only available at age 5–6, so it is impossible to determine how physical activity patterns changed over time. Additionally, while accelerometers can provide a more objective measurement of physical activity than parent-reported data, they have limitations as well. Accelerometers are proficient in capturing overall physical activity and sedentary behaviors, but they lack precision in identifying specific activities. They are unable to provide contextual insights into the motivations behind activity patterns. This limitation may hinder a comprehensive understanding of behaviors drivers. Furthermore, another limitation of this study is the small effect size that was found. All the standard beta coefficients of the significant relationships fell within the range of 0.1 to 0.3. Mental health is a complex construct and it could have been influenced a variety of other factors such as genetics, social conditions and lifestyle [35, 36]. Although physical activity has been shown to have impacts on children’s mental health, it may not be the most dominant factor. As a result, the small effect size of physical activity variables is reasonable. Moreover, the small effect size may also be attributed to the device limitations mentioned earlier, particularly the absence of information about the type and context of physical activity. To gain a better understanding of how physical activity affects mental health in children, future studies should adopt a comprehensive approach that encompasses the types and context of physical activity, along with their interaction with genetic or other lifestyle factors.

## Conclusion

Our research indicated that children who engage in more physical activity at age 5–6 could be likely to experience fewer peer problems at age 10–11. For boys, greater activity levels at age 5–6 could be an indicator of hyperactivity at age 10–11. The findings from this study may help to shed light on the relationships between physical activity in early childhood and mental health in middle childhood. Understanding these relationships between physical activity, sedentary time and mental health may provide new insights on developing effective and targeted strategies from an early age to improve mental health in children.

### Supplementary Information

**Additional file 1.** Supplementary Material.

## Data Availability

The datasets used during the current study are not publicly available due to data restrictions indicated in the informed consent forms but are available from the corresponding author on reasonable request.

## Abbreviations

CPM: Counts Per Minute
LPA: Light Physical Activity
MPA: Moderate Physical Activity
MVPA: Moderate to Vigorous Physical Activity
SDQ: The Strengths and Difficulties Questionnaire
VPA: Vigorous Physical Activity
