# Supplementary material for: A prospective analysis of physical activity and mental health in children: the GECKO Drenthe cohort
Source: Int J Behav Nutr Phys Act. 2023 Sep 25;20:114. doi: 10.1186/s12966-023-01506-1 (PMC10521540; doi:10.1186/s12966-023-01506-1)
Supplement: Supplementary file 1 — Additional file 1. Supplementary Material. [file 12966_2023_1506_MOESM1_ESM.docx]

**Supplementary Material**

**Table S1: Multiple linear regression analysis for MPA, VPA and SDQ subscales (Peer problems, Hyperactivity)**

|  | **Boys** | | | **Girls** | | |
| --- | --- | --- | --- | --- | --- | --- |
|  | **B** | **Std. B** | **95% CI of B** | **B** | **Std. B** | **95% CI of B** |
| ***Peer problems*** | | | | | | |
| **MPA (per 10 min)** | | | | | | |
| Model 1 | -0.397** | -0.126 | (-0.693, -0.100) | -0.325* | -0.118 | (-0.595, -0.056) |
| Model 2 | -0.441** | -0.140 | (-0.747, -0.135) | -0.341* | -0.124 | (-0.617, -0.065) |
| Model 2 + Peer problems at age 5 | -0.495** | -0.156 | (-0.809, -0.182) | -0.262 | -0.094 | (-0.549, 0.026) |
| **VPA (per 10 min)** | | | | | | |
| Model 1 | -0.285** | -0.165 | (-0.447, -0.122) | -0.236** | -0.149 | (-0.391, -0.082) |
| Model 2 | -0.301** | -0.174 | (-0.470, -0.133) | -0.215** | -0.135 | (-0.373, -0.056) |
| Model 2 + Peer problems at age 5 | -0.332** | -0.191 | (-0.505, -0.159) | -0.212** | -0.137 | (-0.374, -0.051) |
| ***Hyperactivity*** | | | | | | |
| **MPA (per 10 min)** | | | | | | |
| Model 1 | 0.950** | 0.158 | (0.384, 1.516) | 0.472* | 0.100 | (0.010, 0.935) |
| Model 2 | 1.073** | 0.178 | (0.502, 1.644) | 0.459 | 0.097 | (-0.018, 0.936) |
| Model 2 + Hyperactivity at age 5 | 0.437 | 0.075 | (-0.070, 0.944) | -0.037 | -0.008 | (-0.442, 0.367) |
| **VPA (per 10 min)** | | | | | | |
| Model 1 | 0.358* | 0.108 | (0.045, 0.671) | 0.086 | 0.032 | (-0.181, 0.354) |
| Model 2 | 0.445** | 0.134 | (0.126, 0.764) | 0.118 | 0.043 | (-0.157, 0.393) |
| Model 2 + Hyperactivity at age 5 | 0.195 | 0.061 | (-0.083, 0.474) | -0.203 | -0.076 | (-0.433, 0.027) |
| **P<0.01, *P<0.05. Model 1: crude model; Model 2: the primary adjusted model, adjusting for age, BMI, family size, maternal education, wear time and season; Model 2+ : further adjusting for SDQ scores at age 5 in addition to model 2. SDQ: the Strengths and Difficulties Questionnaire; MPA: Moderate Physical Activity; VPA: Vigorous Physical Activity. | | | | | | |

**Table S2: Multiple linear regression analysis for PA and SDQ subscales (Emotional problems, Behavioral problems, Prosocial behaviors)**

|  | **Boys** | | | **Girls** | | |
| --- | --- | --- | --- | --- | --- | --- |
|  | **B** | **Std. B** | **95% CI of B** | **B** | **Std. B** | **95% CI of B** |
| ***Emotional problems*** | | | | | | |
| **Physical activity volume (Per 100 cpm)** | | | | | | |
| Model 1 | -0.110 | -0.063 | (-0.274, 0.055) | 0.026 | 0.015 | (-0.143, 0.194) |
| Model 2 | -0.135 | -0.078 | (-0.310, 0.040) | -0.018 | -0.011 | (-0.193, 0.157) |
| Model 2 + Emotional problems at age 5 | -0.093 | -0.053 | (-0.270, 0.085) | 0.006 | 0.003 | (-0.166, 0.178) |
| **MVPA (per 10 min)** | | | | | | |
| Model 1 | -0.150 | -0.044 | (-0.472, 0.173) | -0.056 | -0.016 | (-0.390, 0.278) |
| Model 2 | -0.161 | -0.047 | (-0.499, 0.178) | -0.080 | -0.024 | (-0.423, 0.263) |
| Model 2 + Emotional problems at age 5 | -0.023 | -0.007 | (-0.368, 0.321) | -0.061 | -0.018 | (-0.401, 0.280) |
| **MPA (per 10 min)** | | | | | | |
| Model 1 | -0.183 | -0.047 | (-0.552, 0.186) | 0.019 | 0.005 | (-0.355, 0.392) |
| Model 2 | -0.193 | -0.050 | (-0.578, 0.192) | -0.040 | -0.011 | (-0.423, 0.343) |
| Model 2 + Emotional problems at age 5 | -0.077 | -0.020 | (-0.470, 0.316) | -0.032 | -0.008 | (-0.414, 0.350) |
| **VPA (per 10 min)** | | | | | | |
| Model 1 | -0.080 | -0.037 | (-0.283, 0.124) | -0.099 | -0.045 | (-0.314, 0.116) |
| Model 2 | -0.087 | -0.041 | (-0.300, 0.127) | -0.088 | -0.040 | (-0.308, 0.131) |
| Model 2 + Emotional problems at age 5 | 0.026 | 0.012 | (-0.193, 0.245) | -0.061 | -0.028 | (-0.278, 0.156) |
| **LPA (per 30 min)** | | | | | | |
| Model 1 | -0.134 | -0.091 | (-0.273, 0.005) | 0.066 | 0.047 | (-0.070, 0.202) |
| Model 2 | -0.146* | -0.099 | (-0.290, -0.001) | 0.028 | 0.020 | (-0.118, 0.174) |
| Model 2 + Emotional problems at age 5 | -0.168* | -0.112 | (-0.315, -0.021) | 0.041 | 0.030 | (-0.105, 0.188) |
| **Sedentary time (per 30 min)** | | | | | | |
| Model 1 | 0.549 | 0.067 | (-0.231, 1.328) | -0.352 | -0.041 | (-1.204, 0.501) |
| Model 2 | 0.907 | 0.110 | (-0.005, 1.819) | 0.022 | 0.003 | (-0.921, 0.966) |
| Model 2 + Emotional problems at age 5 | 0.867 | 0.105 | (-0.062, 1.796) | -0.061 | -0.007 | (-0.998, 0.876) |
| ***Behavioral problems*** | | | | | | |
| **Physical activity volume (Per 100 cpm)** | | | | | | |
| Model 1 | 0.048 | 0.040 | (-0.066, 0.162) | 0.014 | 0.014 | (-0.082, 0.110) |
| Model 2 | 0.046 | 0.038 | (-0.076, 0.168) | 0.024 | 0.024 | (-0.076, 0.124) |
| Model 2 +Behavioral problems at age 5 | -0.035 | -0.029 | (-0.153, 0.084) | 0.033 | 0.034 | (-0.068, 0.133) |
| **MVPA (per 10 min)** | | | | | | |
| Model 1 | 0.124 | 0.053 | (-0.100, 0.348) | -0.030 | -0.015 | (-0.220, 0.160) |
| Model 2 | 0.128 | 0.054 | (-0.107, 0.363) | 0.038 | 0.020 | (-0.159, 0.235) |
| Model 2 +Behavioral problems at age 5 | -0.059 | -0.025 | (-0.290, 0.172) | 0.028 | 0.015 | (-0.172, 0.228) |
| **MPA (per 10 min)** | | | | | | |
| Model 1 | 0.178 | 0.066 | (-0.078, 0.434) | -0.012 | -0.005 | (-0.224, 0.201) |
| Model 2 | 0.178 | 0.066 | (-0.089, 0.445) | 0.043 | 0.020 | (-0.176, 0.262) |
| Model 2 +Behavioral problems at age 5 | -0.087 | -0.032 | (-0.353, 0.179) | 0.047 | 0.021 | (-0.178, 0.272) |
| **VPA (per 10 min)** | | | | | | |
| Model 1 | 0.027 | 0.018 | (-0.114, 0.168) | -0.026 | -0.021 | (-0.148, 0.096) |
| Model 2 | 0.032 | 0.021 | (-0.117, 0.180) | 0.024 | 0.020 | (-0.102, 0.150) |
| Model 2 +Behavioral problems at age 5 | -0.029 | -0.019 | (-0.173, 0.116) | 0.016 | 0.013 | (-0.110, 0.142) |
| **LPA (per 30 min)** | | | | | | |
| Model 1 | 0.003 | 0.003 | (-0.094, 0.101) | 0.012 | 0.015 | (-0.065, 0.089) |
| Model 2 | -0.146* | 0.003 | (-0.098, 0.104) | 0.025 | 0.032 | (-0.059, 0.109) |
| Model 2 +Behavioral problems at age 5 | -0.168* | -0.031 | (-0.130, 0.066) | 0.053 | 0.066 | (-0.033, 0.139) |
| **Sedentary time (per 30 min)** | | | | | | |
| Model 1 | -0.258 | -0.045 | (-0.800, 0.284) | -0.356 | -0.072 | (-0.839, 0.128) |
| Model 2 | -0.252 | -0.044 | (-0.887, 0.384) | -0.155 | -0.031 | (-0.695, 0.385) |
| Model 2 +Behavioral problems at age 5 | 0.138 | 0.024 | (-0.485, 0.761) | -0.279 | -0.057 | (-0.830, 0.272) |
| ***Prosocial behaviors*** | | | | | | |
| **PA volume (Per 100 cpm)** | | | | | | |
| Model 1 | -0.011 | -0.008 | (-0.151, 0.129) | -0.009 | -0.007 | (-0.130, 0.112) |
| Model 2 | 0.013 | 0.009 | (-0.135, 0.161) | 0.015 | 0.013 | (-0.111, 0.142) |
| Model 2 +Prosocial behaviors at age 5 | -0.007 | -0.005 | (-0.150, 0.136) | 0.008 | 0.006 | (-0.118, 0.133) |
| **MVPA (per 10 min)** | | | | | | |
| Model 1 | -0.051 | -0.018 | (-0.325, 0.224) | 0.006 | 0.002 | (-0.233, 0.245) |
| Model 2 | 0.007 | 0.003 | (-0.278, 0.292) | -0.015 | -0.006 | (-0.263, 0.234) |
| Model 2 +Prosocial behaviors at age 5 | -0.019 | -0.007 | (-0.297, 0.258) | -0.013 | -0.005 | (-0.259, 0.233) |
| **MPA (per 10 min)** | | | | | | |
| Model 1 | -0.089 | -0.027 | (-0.403, 0.225) | -0.018 | -0.007 | (-0.285, 0.249) |
| Model 2 | -0.045 | -0.014 | (-0.369, 0.279) | -0.043 | -0.016 | (-0.320, 0.234) |
| Model 2 +Prosocial behaviors at age 5 | -0.045 | -0.013 | (-0.362, 0.272) | -0.078 | -0.029 | (-0.354, 0.198) |
| **VPA (per 10 min)** | | | | | | |
| Model 1 | 0.010 | 0.006 | (-0.163, 0.183) | 0.015 | 0.010 | (-0.139, 0.169) |
| Model 2 | 0.058 | 0.032 | (-0.122, 0.238) | 0.007 | 0.004 | (-0.152, 0.166) |
| Model 2 +Prosocial behaviors at age 5 | 0.020 | 0.011 | (-0.156, 0.196) | 0.036 | 0.023 | (-0.122, 0.193) |
| **LPA (per 30 min)** | | | | | | |
| Model 1 | 0.034 | 0.027 | (-0.085, 0.153) | 0.012 | 0.012 | (-0.085, 0.110) |
| Model 2 | -0.146* | 0.029 | (-0.085, 0.158) | 0.018 | 0.018 | (-0.088, 0.124) |
| Model 2 +Prosocial behavior at age 5 | -0.168* | 0.028 | (-0.084, 0.154) | -0.014 | -0.014 | (-0.121, 0.092) |
| **Sedentary time (per 30 min)** | | | | | | |
| Model 1 | -0.199 | -0.029 | (-0.863, 0.465) | 0.263 | 0.043 | (-0.346, 0.871) |
| Model 2 | -0.188 | -0.027 | (-0.958, 0.583) | -0.114 | -0.018 | (-0.795, 0.568) |
| Model 2 +Prosocial behavior at age 5 | -0.115 | -0.016 | (-0.864, 0.633) | 0.055 | 0.009 | (-0.626, 0.737) |
| **P<0.01, *P<0.05. Model 1: crude model; Model 2: the primary adjusted model, adjusting for age, BMI, family size, maternal education, wear time and season; Model 2+ : further adjusting for SDQ scores at age 5 in addition to model 2. SDQ: the Strengths and Difficulties Questionnaire; LPA: Light Physical Activity; MPA: Moderate Physical Activity; VPA: Vigorous Physical Activity; MVPA: Moderate to Vigorous Physical Activity. | | | | | | |

**Table S3: Sensitivity analysis for children with valid weekday and weekend accelerometry data.**

|  | **Boys** | | | **Girls** | | |
| --- | --- | --- | --- | --- | --- | --- |
|  | **B** | **Std. B** | **95% CI of B** | **B** | **Std. B** | **95% CI of B** |
| ***Peer Problems*** | | | | | | |
| **Physical activity volume (per 100 cpm)** | | | | | | |
| Model 1 | -0.190* | -0.135 | (-0.344, -0.035) | -0.129 | -0.094 | (-0.290, 0.031) |
| Model 2 | -0.212* | -0.150 | (-0.377, -0.047) | -0.172* | -0.125 | (-0.337, -0.008) |
| Model 2+ peer problems at age 5 | -0.210* | -0.147 | (-0.380, -0.040) | -0.070 | -0.051 | (-0.231, 0.092) |
| **MVPA (per 10 min)** | | | | | | |
| Model 1 | -0.404** | -0.146 | (-0.708, -0.100) | -0.366* | -0.136 | (-0.677, -0.056) |
| Model 2 | -0.433** | -0.156 | (-0.754, -0.112) | -0.401* | -0.149 | (-0.721, -0.081) |
| Model 2+ peer problems at age 5 | -0.444** | -0.157 | (-0.773, -0.116) | -0.206 | -0.078 | (-0.524, 0.112) |
| **LPA (per 30 min)** | | | | | | |
| Model 1 | -0.016 | -0.014 | (-0.146, 0.114) | -0.005 | -0.005 | (-0.124, 0.115) |
| Model 2 | -0.039 | -0.033 | (-0.174, 0.096) | -0.021 | -0.021 | (-0.150, 0.107) |
| Model 2+ peer problems at age 5 | -0.022 | -0.018 | (-0.163, 0.119) | 0.032 | 0.032 | (-0.092, 0.157) |
| **Sedentary time (per 30 min)** | | | | | | |
| Model 1 | 0.690 | 0.106 | (-0.028, 1.409) | 0.258 | 0.038 | (-0.533, 1.049) |
| Model 2 | 0.948* | 0.145 | (0.101, 1.794) | 0.542 | 0.080 | (-0.303, 1.386) |
| Model 2+ peer problems at age 5 | 0.872 | 0.131 | (-0.003, 1.748) | 0.108 | 0.016 | (-0.718, 0.933) |
| ***Hyperactivity*** | | | | | | |
| **Physical activity volume (per 100 cpm)** | | | | | | |
| Model 1 | 0.423** | 0.154 | (0.122, 0.724) | 0.138 | 0.059 | (-0.135, 0.411) |
| Model 2 | 0.481** | 0.175 | (0.165, 0.798) | 0.121 | 0.052 | (-0.165, 0.406) |
| Model 2+ hyperactivity at age 5 | 0.250 | 0.095 | (-0.023, 0.522) | -0.119 | -0.051 | (-0.365, 0.128) |
| **MVPA (per 10 min)** | | | | | | |
| Model 1 | 0.908** | 0.167 | (0.316, 1.500) | 0.298 | 0.065 | (-0.232, 0.829) |
| Model 2 | 1.018** | 0.188 | (0.405, 1.632) | 0.316 | 0.069 | (-0.242, 0.874) |
| Model 2+ hyperactivity at age 5 | 0.612* | 0.118 | (0.087, 1.137) | 3.259 | 0.000 | (-4.503, 11.022) |
| **LPA (per 30 min)** | | | | | | |
| Model 1 | 0.170 | 0.074 | (-0.084, 0.424) | 0.096 | 0.055 | (-0.107, 0.298) |
| Model 2 | 0.186 | 0.081 | (-0.074, 0.446) | 0.070 | 0.040 | (-0.151, 0.292) |
| Model 2+ hyperactivity at age 5 | 0.136 | 0.061 | (-0.086, 0.358) | 0.053 | 0.031 | (-0.137, 0.244) |
| **Sedentary time (per 30 min)** | | | | | | |
| Model 1 | -1.830* | -0.143 | (-3.228, -0.433) | -0.869 | -0.076 | (-2.208, 0.471) |
| Model 2 | -2.236** | -0.175 | (-3.859, -0.614) | -0.640 | -0.056 | (-2.101, 0.820) |
| Model 2+ hyperactivity at age 5 | -1.532* | -0.125 | (-2.935, -0.130) | -0.027 | -0.002 | (-1.277, 1.224) |
| **P<0.01, *P<0.05. Model 1: crude model; Model 2: the primary adjusted model, adjusting for age, BMI, family size, maternal education, wear time and season; Model 2+ : further adjusting for SDQ scores at age 5 in addition to model 2. SDQ: the Strengths and Difficulties Questionnaire; LPA: Light Physical Activity; MVPA: Moderate to Vigorous Physical Activity. | | | | | | |
